# A Pilot Randomized Controlled Trial of the Family Assessment and Feedback Intervention (FAFI): Effects on Mental Health Literacy and Attitudinal Engagement with Health Supports and Services

**DOI:** 10.1007/s10578-024-01707-0

**Published:** 2024-05-25

**Authors:** Masha Y. Ivanova, Allison Hall, Stanley Weinberger, Sara L. Buckingham, William E. Copeland, Phoenix Crockett, Justin Dainer-Best, Casey D’Alberto, Lauren Dewey, DeShan Foret, Maria Galano, Lisa Goodrich, Lindsay Holly, Nalini Emily Lane, Maureen Leahy, Mathew Lerner, Jasmine Marsh, Ellen W. McGinnis, Melissa Paiva-Salisbury, Judith S. Shaw, Pamela Swift, Rebekah Tinker, James J. Hudziak

**Affiliations:** 1 Department of Psychiatry, University of Vermont, Burlington, VT USA; 2 Department of Pediatrics, University of Vermont, Burlington, VT USA; 3 Department of Psychology, University of Alaska Anchorage, Anchorage, AK USA; 4 Psychology Program, Bard College, Annandale-on-Hudson, NY USA; 5 Department of Psychological Sciences, University of Vermont, Burlington, VT USA; 6 Department of Psychological and Brain Sciences, University of Massachusetts at Amherst, Amherst, MA USA; 7 University of Vermont Medical Center, Burlington, VT USA; 8 Department of Psychology, Marquette University, Milwaukee, WI USA; 9 Children’s Hospital of Philadelphia, Philadelphia, PA USA; 10 Department of Psychology, Coastal Carolina University, Conway, SC USA

**Keywords:** Family assessment and feedback intervention, FAFI, Family-based, Therapeutic assessment feedback, RCT

## Abstract

This randomized controlled trial tested the Family Assessment and Feedback Intervention (FAFI), a new intervention to enhance family engagement with emotional and behavioral health services. The FAFI is a guided conversation with families about results of their multidimensional assessment that is set in the context of motivational enhancement. It differs from other assessment-with-feedback interventions by extending the focus of assessment beyond the target child to parents and the family environment, addressing parental emotional and behavioral problems and competencies, spanning a broad range of children’s and parents’ strengths and difficulties, and being generalizable to many settings and practitioners. Participants were 81 families in primary care pediatrics. The FAFI was associated with a significant increase in parental mental health literacy and with an increase in parental attitudinal engagement with health supports and services that closely approached statistical significance (*p* = .052), while controlling for children’s age and gender and family socioeconomic status.

## Introduction

Despite considerable advances that have been made in prevention and treatment of emotional and behavioral health problems, several comprehensive literature reviews have concluded that poor family engagement with prevention and treatment efforts remains a serious problem [1–4]. Multidimensional and dynamic, family engagement is predicted by many personal, family, logistic, and therapeutic factors [5, 6]. A review of 40 years of research on engagement with children’s mental health services concluded that the five practice elements that were most frequently included in effective engagement interventions were assessment, psychoeducation, soliciting barriers to treatment, promoting treatment accessibility (i.e., removing the barriers), and goal setting [7]. One type of intervention for improving engagement is to provide clients with personalized assessment feedback that compares data about their emotional and behavioral health to normative benchmarks. Assessment feedback interventions typically address individual, family, and therapeutic, but not logistic predictors of engagement, and include the assessment, psychoeducation, and goal setting practice elements.

Several empirically-supported interventions incorporate personalized assessment feedback. Motivational Enhancement Therapy (MET; [8]) combines Motivational Interviewing, a conversation style designed to amplify clients’ disposition for change (MI; [9]), with personalized assessment feedback. Clients are provided with a personalized report about the target behavior (e.g., substance use) that displays their data in relation to normative data (e.g., typical substance use by people their age and gender). When tested in randomized controlled trials (RCTs) with youth, MET reduced social media use and risky sexual behavior and improved response to psychotherapy for youth [10–12].

The Family Check-Up is a landmark application of MI and therapeutic assessment feedback for reducing children’s oppositional and defiant problems [13, 14]. Parents receive feedback about their parenting behavior based on results of a standardized assessment of their interactions with their children, followed by a behavioral parent training intervention, where indicated [14]. Originally developed for prevention of substance abuse in adolescence, the Family Check-Up reduced rates of substance use initiation and growth over the course of middle school and substance abuse symptoms in young adulthood when tested in a school setting [15–17]. When offered to socioeconomically disadvantaged urban families with 2-year-olds, the Family Check-Up was associated with lower children’s disruptive behavior and greater parental involvement when the children were ages three and four [18]. A large, multi-site trial in which it was offered annually to families of toddlers ages two to five demonstrated that it was associated with slower growth of children’s externalizing behavior problems, and lower children’s externalizing behavior problems in primary school [19, 20].

### The Family Assessment and Feedback Intervention

The present study tested the efficacy of a new therapeutic assessment feedback intervention, the Family Assessment and Feedback Intervention (FAFI; [21]). The FAFI is a guided conversation with family members about results of their assessment of both parent and child emotional and behavioral health, family relationships, and the family environment in the context of motivational enhancement. The FAFI aims to help family members to “meet themselves” individually and as a family by identifying areas of similarity and differences in emotional and behavioral profiles between family members and exploring how they affect the family environment, including family relationships and health-related practices.

The FAFI was created to facilitate family engagement with children’s treatment for emotional and behavioral problems. It was intended for implementation at any stage of the treatment process. The FAFI can be delivered at the beginning of the intervention to help establish rapport with the family and facilitate collaborative treatment planning. It can also be implemented after some treatment work has been done, to deepen family members’ understanding of themselves and each other and plan next steps.

While supporting families in their exploration of their data, the clinician weaves in several key concepts of mental health literacy, such as the familial nature of mental health and individual differences in profiles of mental health. The FAFI emphasizes mental health literacy, because while it is essential for the identification and help seeking for emotional and behavioral problems, population rates of mental health literacy are low around the globe [22]. This conversation occurs in the context of motivational enhancement, using techniques from Motivational Interviewing [9] and values-based techniques developed for the FAFI [21].

The FAFI differs from other assessment-with-feedback interventions by extending the focus of assessment beyond the target child to parents and the family environment. Parental emotional and behavioral problems and competencies are addressed by the FAFI directly and comprehensively. Another innovation of the FAFI is that it spans a broad spectrum of strengths and difficulties, for both parents and children. It is based on a broad, empirically-derived taxonomic framework of emotional and behavioral problems that is backed by thousands of studies conducted over decades of research [23]. The FAFI takes approximately an hour.

### The Present Study

The present study was conducted as part of a larger RCT that tested a comprehensive family-based intervention, the Vermont Family Based Approach (VFBA), in a pediatric primary care setting [24–26]. The FAFI was administered immediately after recruited families completed the baseline assessment protocol and before any programmatic offerings of the larger trial of the VFBA. Because the FAFI emphasizes mental health literacy and other assessment feedback interventions increase treatment engagement (e.g., [27–29]), we tested the effects of the FAFI on parental mental health literacy and treatment engagement. The FAFI was tested with community families who were not seeking mental healthcare for their children. Given this, we operationalized engagement as dispositional or attitudinal engagement with health promotion and healthcare services that are focused on emotional and behavioral health.

## Method

### Participants

Participants were 81 children and families recruited at a University of Vermont (UVM) Medical Center primary care pediatric clinic in Burlington, Vermont. The child sample was primarily of preschool age (*M* = 4.89; *SD* 1.88; range = 3–14 years old) and was 46% female (37 girls). Children’s racial/ethnic backgrounds were as follows: 10 (12.3%) African, 5 (6.2%) Arab/Middle Eastern, 6 (7.4%) Asian, 3 (3.7%) Hispanic, 53 (65.4%) White, and 4 (4.9%) Mixed. Family socioeconomic status was split evenly between low-, middle-, and high-earning families [26]. Fifteen families identified as immigrant (18.5%). Twenty six families (32%) identified as single-parent households.

### Setting and Procedures

Families were recruited during non-acute visits by pediatric practitioners. Families completed the baseline assessment after consenting for participation, and were randomized immediately thereafter (41 FAFI; 40 Control). The FAFI was based on results of the baseline assessment, and delivered to the intervention group within the 1st month after completing the baseline assessment. The post-FAFI reassessment was done immediately after the FAFI session with the family. Therefore, the only contact from study staff that participants received in the first 4 weeks of the study were phone calls to schedule the FAFI visit and post-FAFI assessment (FAFI group) or just the assessment (Control group). When asked to take part in the FAFI, families were invited to a conversation about the results of their initial family assessment. They were told that during this conversation they would collaboratively join the research staff in making sense of the data to better understand their family. They were also told that the results would focus on the emotional and behavioral health and wellbeing of individual family members and their family as a whole. For the Control group that did not receive the FAFI, the parallel reassessment was conducted during the 4th week after the baseline assessment (i.e., the same interval as the reassessment for the FAFI group).

### Assessment Instruments

#### Assessment Instruments Used in the FAFI

During the FAFI, personalized graphic profiles of each family’s normatively-referenced data obtained with the following assessment instruments were shared with families.

##### Children’s and Parents’ Emotional and Behavioral Health

We used assessments that are part of the Achenbach System of Empirically Based Assessment (ASEBA; [30]). For preschoolers, we used the Child Behavior Checklist for Ages 1.5–5 completed by parental figures and the Caregiver-Teacher Report Form completed by caregivers or teachers (CBCL/1.5–5 and CTRF [31]). For school-aged children, we used the Child Behavior Checklist for Ages 6–18, Youth Self-Report, and Teacher’s Report Form (CBCL/6–18, YSR, TRF; [32]). For parents, we used the Adult Self-Report and Adult Behavior Checklist, which are self- and collateral-report questionnaires, respectively (ASR, ABCL [33]). ASEBA forms are normed on a representative U.S. national household sample. Factor analyses of their problem items have yielded syndromes that have been supported by confirmatory factor analyses of tens of thousands of children and adults in dozens of societies [34–41]. All scales have good psychometric properties, and multicultural norms are available in addition to U.S. norms. Indicating the overall level of emotional and behavioral problems, mean Total Problems scale scores fell in the normal range for both parents and children in both study arms, as indicated by the CBCL and ASR *t*-scores (FAFI: CBCL *t* = 49.61 (13.86), ASR *t* = 45.26 (12.34); Control: CBCL *t* = 52.11 (12.59), ASR *t* = 45.70 (12.91)).

##### Family Environment and Relationships

We used the Family Environment Scale (FES; [42]) scales of Cohesion, Conflict, Intellectual-Cultural Orientation, Active-Recreational Orientation, Moral-Religious Emphasis, Organization, and Control. FES scales demonstrate strong internal consistency (Cronbach’s α = 0.67–0.79), test–retest reliability (*r* = 0.76–0.86), and construct validity [42].

#### Assessment Instruments Used for Outcome Assessment

Both outcome assessment instruments were developed for the study because psychometrically validated instruments assessing mental health literacy and engagement from the family perspective and in the context of both health promotion and intervention were not available at the time of the study.

##### Parental Mental Health Literacy

The Parental Mental Health Literacy Questionnaire (PMHLQ) is a 23-item self-report questionnaire. It is based on Jorm’s multidimensional conceptualization of mental health literacy as knowledge and beliefs about emotional and behavioral health that aid in its promotion and in the recognition and treatment of emotional and behavioral problems [43]. The questionnaire assesses parental recognition of emotional and behavioral problems (e.g., “I can recognize when someone is suffering from an emotional or behavioral problem”), knowledge about their risk factors and causes (e.g., “Emotional and behavioral problems are caused in part by biological factors (e.g., genes)”), knowledge about the promotion of emotional and behavioral health (e.g., “Taking care of my own emotional and behavioral health is one of the best ways to support my child’s emotional and behavioral health”), knowledge that facilitates appropriate help seeking (e.g., “It is best to get help quickly and not wait”), and knowledge supporting help seeking (e.g., “I know where to look for accurate information about emotional and behavioral problems and their treatment”).

##### Parental Engagement

The Parental Health Engagement Questionnaire (PHEQ) is a 36-item self-report questionnaire that assesses dispositional or attitudinal engagement with family-based activities and services that promote emotional and behavioral health. The PHEQ includes questions assessing (1) recognition of the importance of health-promoting behaviors and practices (e.g., “I believe that health promotion (e.g., healthy nutrition…) is important to family health”), (2) knowledge of the health promoting behaviors and practices (e.g., “I know what food is healthy”), (3) skills for carrying out the behaviors and practices (e.g., “I know how to make healthy food for my family”), and (4) active engagement in health-promoting behaviors and practices (e.g., “I make healthy food for my family”).

### The FAFI

Before the FAFI, the clinician decides who will be invited to the conversation. Family members who take part in the FAFI need to be able to comprehend the concepts introduced in the conversation, regulate their emotions while discussing personal topics, and prioritize children’s needs while discussing family matters if the children are present. Topics that can be disturbing or unsettling for children (e.g., parental substance misuse and traumatic history, financial difficulties) are addressed with the parents without the children. Children ages 7 and younger generally do not participate in the FAFI, children ages 7–11 are invited to some parts of the conversation that can be beneficial to them (e.g., ideas of mental health literacy presented in a developmentally accessible language), and typically developing youth ages 12 and older generally can participate in the full conversation. Ivanova [21] provides more strategies for setting up productive conversations with families. While the FAFI can be carried out with any primary caregivers (e.g., grandparents, aunts/uncles), all adult participants in this study were children’s biological parents, so we will refer to them as “parents” in the following.

The FAFI is a guided conversation in four parts. In the *Introduction*, the clinician introduces several concepts of mental health literacy, including the familial nature of mental health (including the importance of parental mental health for children’s health), individual differences in mental health (i.e., that we are not all created equal with respect to our emotional and behavioral strengths and vulnerabilities), etiological factors predicting mental health problems (i.e., that biology and environment transact over time), and the importance of multi-informant perspectives in assessment. The family is then asked to come up with the *Central Question* to be answered in the session. In community settings, the question is usually framed in terms of greater self-understanding for each family member, and of how the family works together. In clinical settings, this segment is used as an opportunity to shift the family’s framing of the presenting problem from focusing on the target child to considering the entire family. Family members are presented with an *Assessment Summary*, in which their data are plotted in relation to data for normative samples. The Assessment Summary highlights areas of relative strengths and difficulties and is organized around the topics of child’s and parents’ adaptive functioning, emotional and behavioral problems, family relationships, and the family environment. Family members are encouraged to explore how the similarities and differences in their profiles of emotional and behavioral strengths and difficulties affect family relationships and routines. The motivational enhancement techniques used in this conversation are Motivational Interviewing [9] and values-based techniques developed for the FAFI. The FAFI ends with the *Summary* of the main health literacy themes and the family’s results. Where appropriate, a range of family support options are also presented and discussed with the family.

### Training in the FAFI

The FAFI was delivered by project staff, including graduate students in mental health (i.e., Clinical Psychology, Social Work, and Mental Health Counseling), a public health professional, and the senior author, who is a Licensed Clinical Psychologist (MI). The senior author provided a group training to project staff, which addressed the FAFI’s conceptual foundations, interpretation of the personalized graphic profile report provided to each family, and step-by-step session procedures. She also led several FAFI sessions with families in the presence of project staff before they carried them out independently. Before each FAFI that was carried out by project staff independently, the senior author reviewed the family’s personalized graphic profile report with the project staff, emphasizing ways in which the delivery of the intervention would need to be adapted based on the sociodemographic characteristics of the family (e.g., parental educational level and occupation, immigration status, ethnic and racial background).

## Results

Using general linear modeling (GLM) repeated measures analyses, we compared rates of change in parental ratings of mental health literacy and engagement with family health supports and services from baseline assessment to the 4th week after recruitment, which was immediately after the FAFI intervention for the intervention group. We specified a full factorial model with child gender, age, and family SES as covariates.

As Table 1 shows, receiving the FAFI was associated with significantly different rates of change for mental health literacy between the two groups. For ratings of attitudinal engagement with family health supports and services, the difference in the rates of change between the FAFI and Control groups approached statistical significance (*p* = 0.052).

**Table 1:** Results of general linear modeling repeated measures analyses

Predictor	Mean square	*F*	*p*
I. Predicting mental health literacy			
Age	168.81	1.37	.25
SES	980.66	7.94	.01
Gender	852.88	6.91	.01
Group	18.55	.15	.70
Time	16.89	.39	.53
Time*age	44.08	1.03	.32
Time*SES	.95	.02	.88
Time*gender	96.00	96.00	2.23
Time*group	207.22	4.80	.03
II. Predicting engagement with health supports and services			
Age	388.64	1.19	.28
SES	957.38	2.94	.09
Gender	49.52	.15	.70
Group	7.33	.022	.88
Time	49.39	.46	.50
Time*age	30.83	.29	.59
Time*SES	4.68	.04	.84
Time*gender	16.12	.15	.70
Time*group	420.98	3.96	.05

Group = FAFI vs. Control; time = time of assessment: baseline vs. 4 weeks; results are abbreviated

**Fig. 1:**
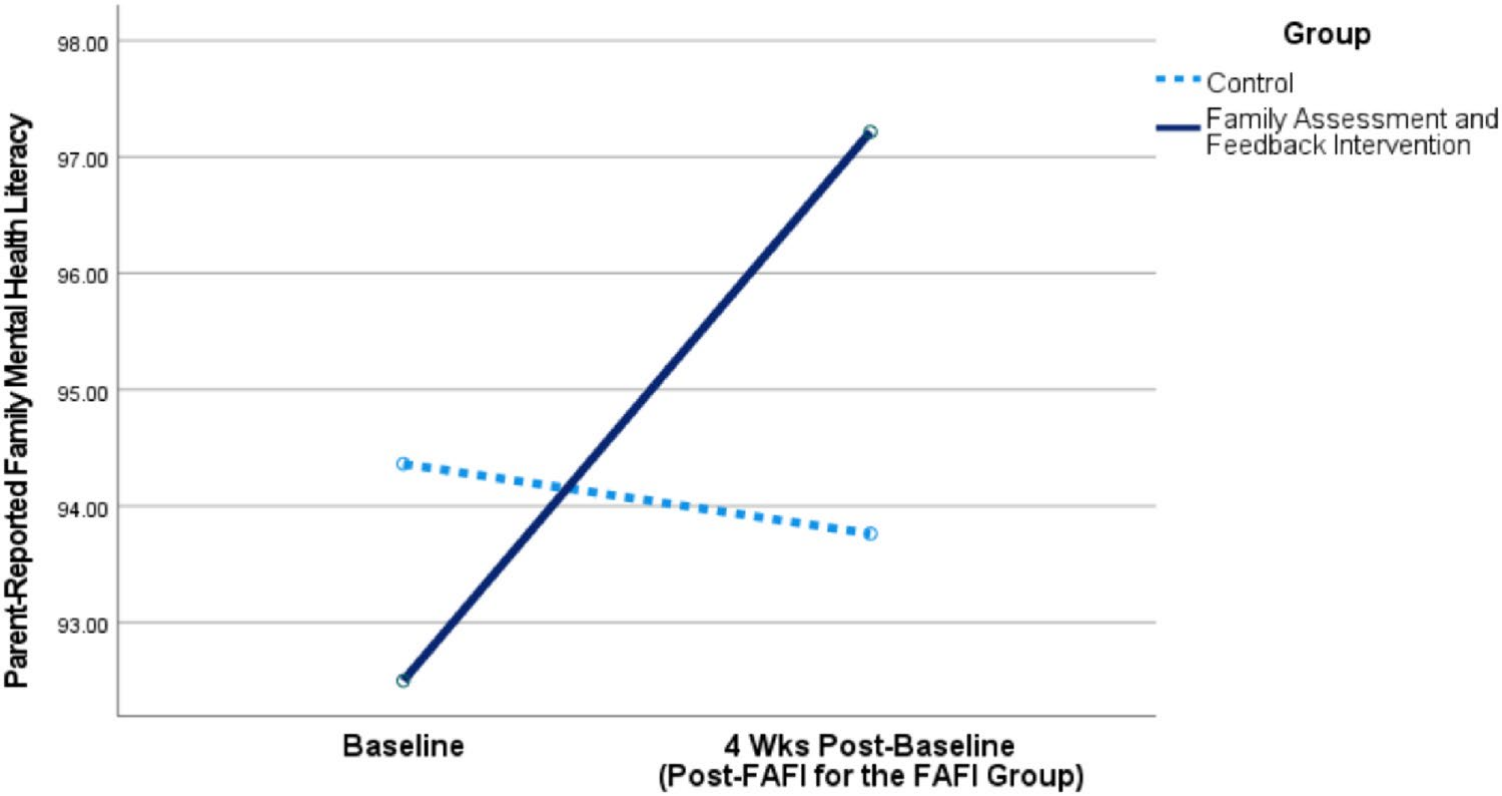
Rates of change of parental mental health literacy for the FAFI and control groups, while controlling for child age and gender and family socioeconomic status

Figure 1 plots the significant interaction for parental mental health literacy, adjusted for child age and gender and family SES. Table 2 presents estimated marginal means for parental mental health literacy and attitudinal engagement with health supports and services at baseline and 4-week assessments, separately for the FAFI and Control Groups.

**Table 2:** Estimated marginal means for parental mental health literacy and engagement with health supports and services at baseline assessment and 4 weeks later

Group	PMHLQ-baseline *M* (SE)	PMHLQ-4 weeks after baseline *M* (SE)	PHEQ-baseline *M* (SE)	PHEQ-4 weeks after baseline *M* (SE)
FAFI	92.50 (1.92)	97.22 (1.28)	156.85 (2.97)	160.14 (2.23)
Control	94.36 (2.01)	93.76 (1.34)	160.13 (3.12)	155.85 (2.34)

Means are adjusted for the model covariates (child age, gender, and family SES)

*PMHLQ* parental mental health literacy questionnaire, *PHEQ* parental health engagement questionnaire

## Discussion

The FAFI is a guided conversation that explores topics of emotional and behavioral health literacy and helps family members to better understand their profiles of emotional and behavioral health, including how they contribute to family relationships. The FAFI introduces several key concepts of mental health literacy, such as descriptions of a broad range of possible emotional and behavioral strengths and difficulties, the idea of individual differences in profiles of emotional and behavioral strengths and difficulties, and how to detect significant problems and obtain competent care. Although these concepts may appear self-evident to health professionals, they are often unfamiliar to family members. Reviewing them with family members and exploring their emotional and behavioral health profiles can help promote self and mutual understanding and help parents to take responsibility for their emotional and behavioral health.

Given the FAFI’s emphasis on mental health literacy, it is not surprising that it was associated with significant improvements in mental health literacy in this study. We were also not surprised by our finding that the FAFI was associated with increases in parental attitudinal engagement with health supports and services that closely approached statistical significance. Although we lacked statistical power to test parental mental health literacy as a mechanism through which it affected engagement, we suspect that it served as such a mechanism because others have found that parental mental health literacy predicted family engagement with children’s mental health care over time. Yeh et al. [44] found that parental beliefs in the biopsychosocial etiologies of mental health problems predicted utilization of child mental health services over a 2-year period, while parental beliefs consistent with other etiologies (e.g., sociological, spiritual, and nature disharmony) did not. Similarly, Mendenhall [45] reported that parental understanding of children’s mood disorders (i.e., their etiology, clinical presentation, and course) and of available treatments for these disorders prior to treatment predicted treatment engagement.

An important consideration is the practicality of the FAFI in primary care as an intervention that can facilitate early detection of emotional and behavioral problems for both children and parents and that can enhance parental felt responsibility for the quality of the family environment, the FAFI holds promise in primary care. However, the complex issues affecting current systems of primary care result in the organizational emphasis on patient throughput and efficiency, making it unlikely that even the most willing primary care practitioner would spend an hour with a family. Given this, the FAFI may be best delivered by experienced nurses or co-located mental health professionals. Notably, when implemented in a primary care setting, an adaptation of the Family Checkup for the treatment of children’s overweight was delivered by paraprofessionals and professionals in “behavioral health, social work, public health, exercise science, and health promotion” [46], p. 467.

Our results should be interpreted in the context of the limitations of this trial. Although the FAFI was the first intervention that was offered to families in the intervention group after their baseline assessment, this trial was embedded in a trial of a comprehensive family-based intervention [25, 26]. Participants’ expectancies about the subsequent intervention could have affected their ratings of engagement. Another limitation is that the effects of the FAFI were measured immediately after the intervention, so it is not clear whether they were sustained. The outcomes were also measured solely by parental reports and by new assessment instruments that were developed for this trial. Although all project staff were trained and supervised by the author of the FAFI (MI), we did not formally assess intervention fidelity and staff performance during the FAFI.

Despite these limitations, results of this trial are encouraging, considering that the FAFI is a one-session intervention that is generally completed within one hour. We are further encouraged that its efficacy was demonstrated with community families where children were not experiencing significant emotional and behavioral problems, a known predictor of parental help seeking (e.g., [5]). Also, although we lacked data to formally compare the fidelity and performance for staff of different backgrounds, the FAFI was delivered by a wide range of project staff, including graduate students in Clinical Social Work or Mental Health Counseling, a public health professional, and master’s- and doctoral-level mental health clinicians. Given such variety of practitioners who can deliver the FAFI, its short length, and breadth of addressed topics, it should be generalizable to a variety of settings and practitioners.

## Summary

This randomized controlled trial tested the Family Assessment and Feedback Intervention (FAFI) to enhance family engagement with emotional and behavioral health supports and services. The FAFI is an assessment-with-feedback intervention that differs from other assessment-with-feedback interventions by extending the focus of assessment beyond the target child to parents and the family environment, addressing parental emotional and behavioral problems and competencies, spanning a broad range of children’s and parents’ strengths and difficulties, and being generalizable to many settings and practitioners. The FAFI introduces several key concepts of mental health literacy, such as descriptions of a broad range of possible emotional and behavioral strengths and difficulties, the idea of individual differences in profiles of emotional and behavioral strengths and difficulties, and how to detect significant problems and obtain competent care. Reviewing them with family members and exploring their emotional and behavioral health profiles can help promote self and mutual understanding and help parents to take responsibility for their emotional and behavioral health. Eighty-one families were recruited in a primary care pediatrics office. The FAFI was associated with a significant increase in parental mental health literacy and with an increase in parental engagement with health supports and services that closely approached statistical significance (*p* = .052), while controlling for children’s age and gender and family socioeconomic status. The FAFI is a short intervention that can be delivered by a wide range of practitioners across a variety of settings.

## Data Availability

The authors will consider requests for data sharing, which should be sent to the corresponding author.
